# Supplementary material for: LRRC59 cooperates with nuclear transporters to restrain the nuclear envelope repair machinery and safeguard genome integrity
Source: Nat Commun. 2025 Dec 12;16:11211. doi: 10.1038/s41467-025-65994-4 (PMC12715200; doi:10.1038/s41467-025-65994-4)
Supplement: Supplementary file 1 — Supplementary Information [file 41467_2025_65994_MOESM1_ESM.pdf]

Supplementary Information for:

LRRC59 cooperates with nuclear transporters to restrain the nuclear envelope repair machinery and safeguard genome integrity

Romy Timmer, Aurélie Bellanger, Sarah Peeters, Hera Kim, Laura Rodriguez de la Ballina, Sissel Eikvar, Annemijn J. Arns, Esmée Oortgijs, Nikolina Sekulić, Winnok H. De Vos, and Coen Campsteijn

This PDF contains:
- Supplementary Figures 1-10
- Supplementary Tabl 1

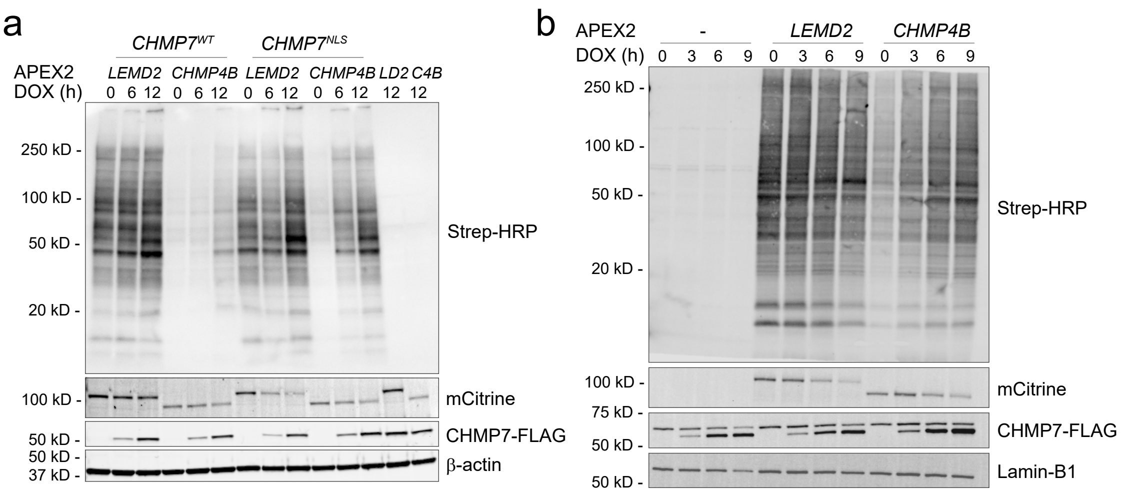

Supplementary figure 1. Validation of APEX2-LEMD2 and -CHMP4B labeling upon CHMP7 induction
A Western blot analysis of biotinylated proteins in RPE1 LEMD2-APEX2-mCitrine and CHMP4B-APEX2-mCitrine cell lines, treated with DOX for 0, 6, or 12 hours to induce CHMP7^{WT}-FLAG or CHMP7^{NLS}-FLAG alleles. Biotinylated proteins were detected using a streptavidin-HRP conjugate, with β-actin used as a loading control. B Western blot analysis of biotinylated proteins in parental RPE1, RPE1 LEMD2-APEX2-mCitrine, and RPE1 CHMP4B-APEX2-mCitrine cell lines, treated with DOX for 0, 3, 6, or 9 hours to induce the CHMP7^{NES*}-FLAG allele. Biotinylated proteins were detected using a streptavidin-HRP conjugate, with Lamin-B1 used as a loading control.

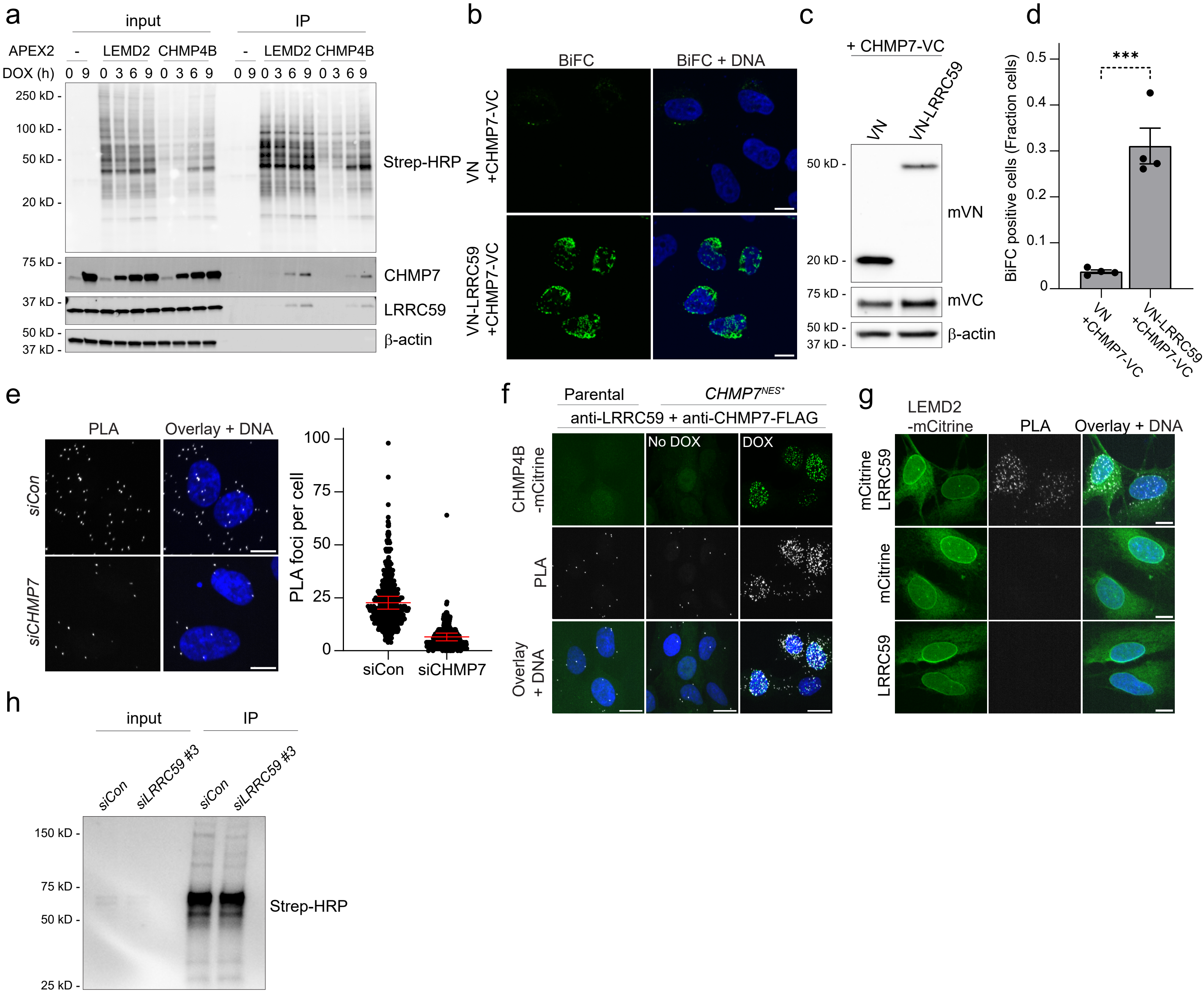

Supplementary figure 2. Identification of LRRC59 as a LEMD2-CHMP7 interacting protein
A LRRC59 is enriched in streptavidin pulldowns after CHMP7^{NES*} induction. Western blot of input and IP fractions of RPE1 LEMD2-APEX2-mCitrine or CHMP4B-APEX2-mCitrine cells after 0, 3, 6, or 9 hours of CHMP7^{NES*} induction and biotin labelling. Biotinylated proteins were detected using streptavidin-HRP, CHMP7 and LRRC59 identified using specific antibodies and β-actin was used as a loading control. B-D LRRC59 and CHMP7 interact at the ER. B, representative confocal images of HeLa K cells co-transfected with CHMP7^{WT}-mVC and mVN-LRRC59 or a negative control (mVN) split mVenus fusions. Cells were stained for DNA with Hoechst. Scale bars, 10 μm. C, Western blot analysis of transgene expression levels, with β-actin used as a loading control. D, Bar graph showing fraction of cells positive for BiFC signal. Error bars: mean ± SEM, 4 independent experiments; n = 207 (mVN + CHMP7-mVC); 223 cells (mVN-LRRC59 + CHMP7-mVC). ***P = 0.0004, two-tailed unpaired Student's t-test. E Endogenous LRRC59 and CHMP7 colocalize at the ER. Representative confocal images of PLA in RPE1 cells with antibodies against LRRC59 and CHMP7 as indicated, with siRNA-depletion of CHMP7 included as specificity control. DNA was stained with Hoechst. Scale bars, 10 μm; N = 3. F LRRC59 colocalizes with NE repair proteins upon CHMP7^{NES*} induction. Representative confocal images of RPE1 CHMP4B-APEX2-mCitrine parent cells or cells expressing doxycycline-inducible CHMP7NES*, incubated with doxycycline to induce CHMP7^{NES*} expression. PLA with antibodies against LRRC59 and FLAG-tag (targeting exogenous CHMP7^{NES*}), and DNA was stained with Hoechst. Scale bars, 20 μm; N = 3. G Representative confocal images of PLA quantification in Figure 3D. DNA was stained with Hoechst. Scale bars, 10 μm; N = 3. H The ER luminal domains of LRRC59 and LEMD2 are in close proximity. Western blot of input and IP fractions from control and LRRC59-depleted RPE1 LRRC59-TurboID cells after biotin labeling. Biotinylated proteins were detected using a streptavidin-HRP conjugate. Source data are provided as a Source Data file.

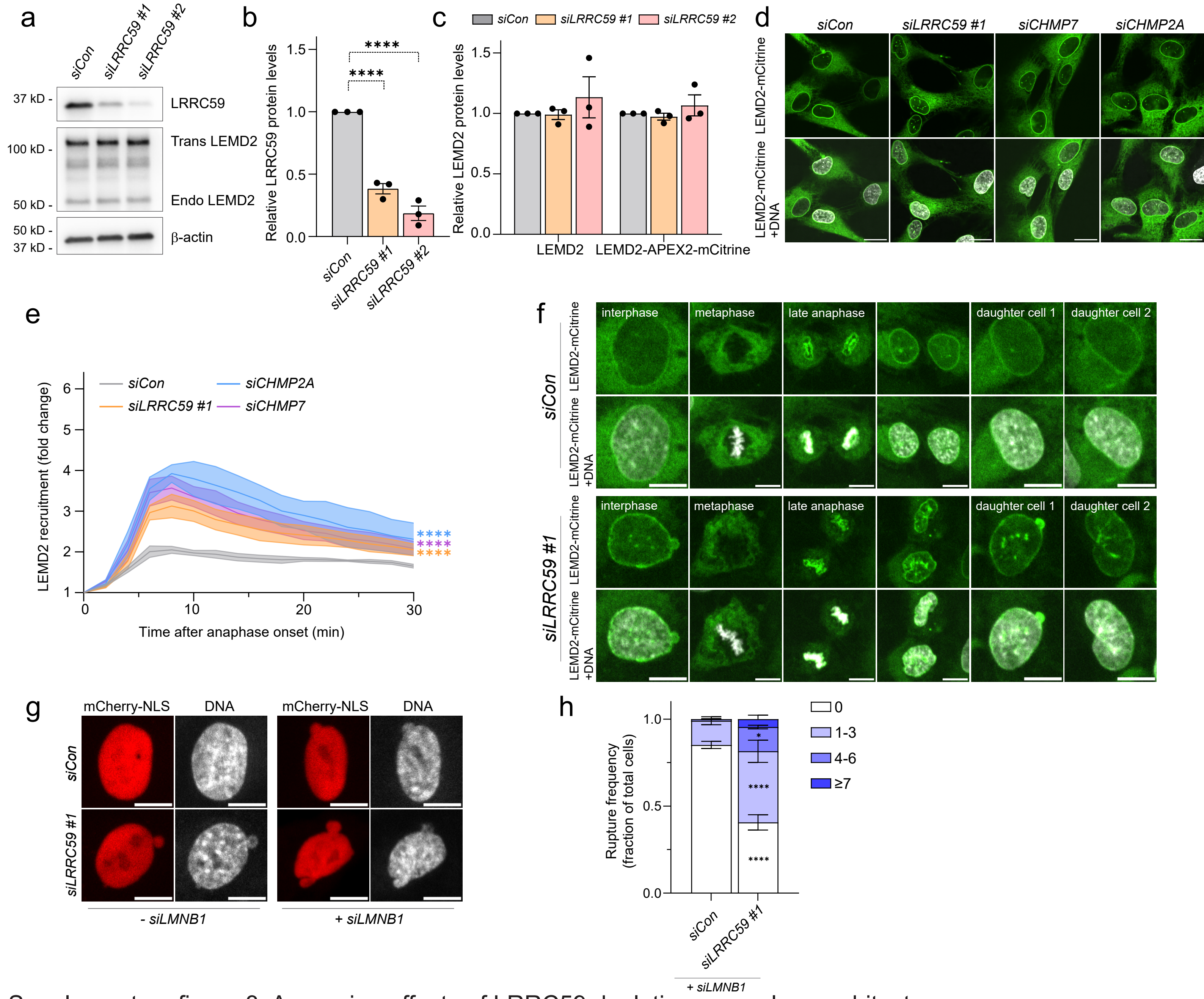

Supplementary figure 3. Assessing effects of LRRC59 depletion on nuclear architecture

A Western blot showing LRRC59 and LEMD2 protein levels after LRRC59 depletion using siRNAs #1 and #2 in RPE1 LEMD2-APEX2-mCitrine mCherry-NLS cells using specific antibodies and β-actin was used as a loading control. B Quantification of relative protein levels of endogenous LRRC59 after LRRC59 depletion. siCon vs siLRRC59 #1, ****$P < 0.0001$; siCon vs siLRRC59 #2, ****$P < 0.0001$. One-way ANOVA with Dunnett's test. N = 3. C Quantification of relative protein levels of endogenous LEMD2 and transgenic LEMD2-APEX2-mCitrine after LRRC59 depletion. D Intranuclear LEMD2 tubules in cells depleted of ESCRT-III components mirror LRRC59 knockdown. Representative live-cell confocal images of RPE1 LEMD2-APEX2-mCitrine cells treated with indicated siRNAs. DNA was stained using SPY-650. Scale bars, 20 μm; N = 3. E Quantification from live-cell imaging of RPE1 LEMD2-APEX2-mCitrine cells with indicated siRNAs at indicated timepoints after anaphase onset (t = 0 min). Error bars: mean ± SEM, 3 independent experiments; n = 42 (siCon); 45 (siLRRC59 #1); 52 (siCHMP7); 41 cells (siCHMP2A). siCon vs siLRRC59 #1, ****$P < 0.0001$; siCon vs siCHMP7, ****$P < 0.0001$; siCon vs siCHMP2A, ****$P < 0.0001$. One-way ANOVA with Dunnett's test. F Elevated LEMD2 levels during NE reformation culminate in LEMD2 nuclear tubules in interphase. Stills from live-cell imaging of RPE1 LEMD2-APEX2-mCitrine cells treated with either siCon or siLRRC59 #1. DNA was stained using SPY-650. Scale bars, 10 μm; N = 3. G Representative confocal images of nuclear herniations observed after (simultaneous) knockdown of LRRC59 and LMNB1 in RPE1 CHMP4B-LAP-mNG mCherry-NLS cells. DNA was stained using SPY-650. Scale bars, 10 μm; N = 3 or 4. H Quantification of rupture frequency of RPE1 CHMP4B-LAP-mNG mCherry-NLS cells treated with either siLMNB1+siCon or siLMNB1+siLRRC59 #1 represented as the fraction of total cells. Error bars: mean ± SEM, 3 independent experiments; n = 445 cells (siLMNB1+siCon); 403 (siLMNB1+siLRRC59 #1). 0: siLMNB1+siCon vs siLMNB1+siLRRC59 #1, ****$P < 0.0001$. 1-3: siLMNB1+siCon vs siLMNB1+siLRRC59 #1, ****$P < 0.0001$. 4-6: siLMNB1+siCon vs siLMNB1+siLRRC59 #1, *$P = 0.0308$. ≥7: siLMNB1+siCon vs siLMNB1+siLRRC59 #1, NS, $P = 0.7651$. One-way ANOVA with Šidák's test. Source data are provided as a Source Data file.

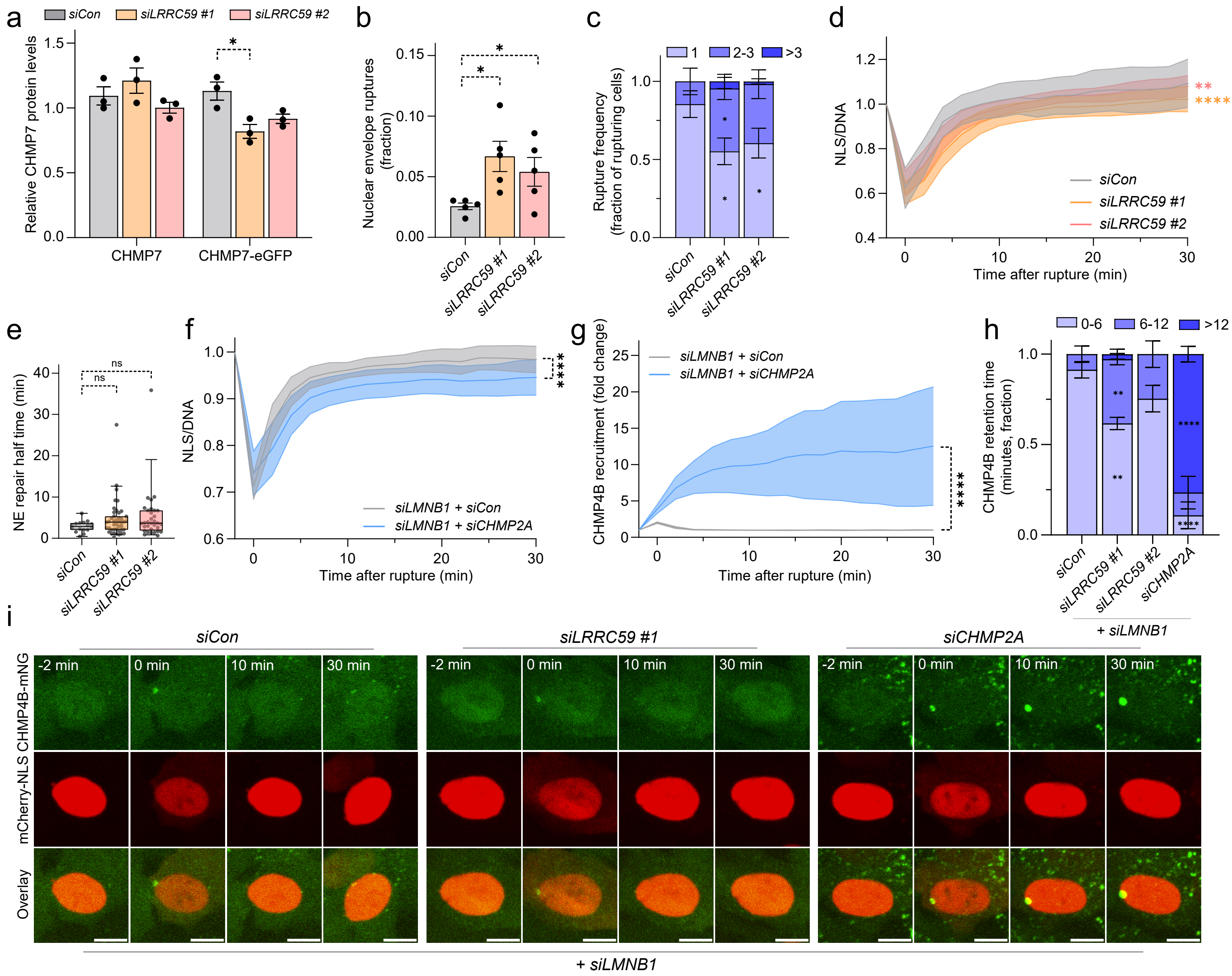

Supplementary figure 4. Consequences of LRRC59 depletion on NE stability and repair
A Quantification of relative protein levels of endogenous CHMP7 and transgenic CHMP7-eGFP post-LRRC59 depletion. siCon vs siLRRC59 #1, *P = 0.0135. Two-way ANOVA with Tukey's test. B-C LRRC59 depletion increases NE rupture frequency. B, Live-cell imaging of RPE1 CHMP4B-LAP-mNG mCherry-NLS cells treated with siCon or siLRRC59 followed by quantification of the fraction of cells experiencing NE rupture. Error bars: mean ± SEM, 5 independent experiments, with dots representing the mean per experiment; n = 1161 (siCon); 824 (siLRRC59 #1); 1211 cells (siLRRC59 #2). siCon vs siLRRC59 #1, *P = 0.0278; siCon vs siLRRC59 #2, *P = 0.0452. One-way ANOVA with Fisher's LSD test. C, as panel B but followed by quantification of the rupture frequency of each rupturing cell. Error bars: mean ± SEM, 3 independent experiments; n = 16 (siCon); 32 (siLRRC59 #1); 34 cells (siLRRC59 #2). 1: siCon vs siLRRC59 #1, *P = 0.0117; siCon vs siLRRC59 #2, *P = 0.0385. 2-3: siCon vs siLRRC59 #1, *P = 0.0327; siCon vs siLRRC59 #2, NS, P = 0.0558. ≥4: siCon vs siLRRC59 #1, NS, P = 0.869; siCon vs siLRRC59 #2, NS, P = 0.9786. One-way ANOVA with Dunnett's test. D LRRC59 depletion has marginal effects on NE repair kinetics. Quantification of nuclear mCherry-NLS over time in RPE1 CHMP4B-LAP-mNG mCherry-NLS cells treated with indicated siRNAs. Error bars: mean (lines) ± 95% CI (bands), 5 independent experiments; n = 19 (siCon); 44 (siLRRC59 1); 32 cells (siLRRC59 #2). siCon vs siLRRC59 #1, ****P < 0.0001; siCon vs siLRRC59 #2, **P = 0.0012. One-way ANOVA of AUC with Dunnett's test. E Quantification of nuclear re-accumulation halftime of mCherry-NLS from panel D. Box, 25-75% percentile; whiskers, 5–95% percentile; medians, 2.87 min (siCon), 3.91 min (siLRRC59 #1) and 3.65 min (siLRRC59 #2); dots representing the halftime per rupture, 5 independent experiments; n = 19 (siCon); 44 (siLRRC59 1); 32 cells (siLRRC59 #2). siCon vs siLRRC59 #1, NS, P = 0.2323; siCon vs siLRRC59 #2, NS, P = 0.1641. Kruskal-Wallis with Dunn's test. F CHMP2A depletion has marginal effects on mCherry-NLS kinetics during NE rupture-repair cycles. Quantification of nuclear mCherry-NLS over time in RPE1 CHMP4B-LAP-mNG mCherry-NLS cells treated with indicated siRNAs. Error bars: mean (lines) ± 95% CI (band), 3 independent experiments; n = 50 (siLMNB1 + siCon); 21 cells (siLMNB1 + siCHMP2A). siLMNB1 + siCon vs siLMNB1 + siCHMP2A, ****P

Supplementary figure 4 continued...

< 0.0001. Two-tailed unpaired Student's t-test of AUC. G CHMP2A depletion abrogates ESCRT-III function at NE rupture sites. Quantification of CHMP4B-LAP-mNG intensities at NE rupture sites. Error bars: mean (lines) ± 95% CI (bands), 3 independent experiments; n = 53 (siLMNB1 + siCon); 29 cells (siLMNB1 + siCHMP2A). siLMNB1 + siCon vs siLMNB1 + siCHMP2A, ****P < 0.0001. Two-tailed unpaired Student's t-test of AUC. H-I CHMP4B retention time at rupture site is unaltered upon LRRC59 depletion. H, quantification of CHMP4B-LAP-mNG retention time based on Figure 5D and Supplementary figure 4G. Error bars: mean ± SEM, 3 independent experiments; n = 54 (siCon + siLMNB1); 43 (siLRRC59 #1 + siLMNB1); 59 (siLRRC59 #2 + siLMNB1); 27 cells (siCHMP2A + siLMNB1). 0-6: siCon vs siLRRC59 #1, **P = 0.0015; siCon vs siCHMP2A, ****P <0.001. 6-12: siCon vs siLRRC59 #1, **P = 0.0038. >12: siCon vs siCHMP2A, ****P <0.001. Two-way ANOVA with Dunnett's test. I, representative stills from live-cell imaging of RPE1 CHMP4B-LAP-mNG mCherry-NLS cells treated with indicated siRNAs. Scale bars, 10 μm; N = 3.

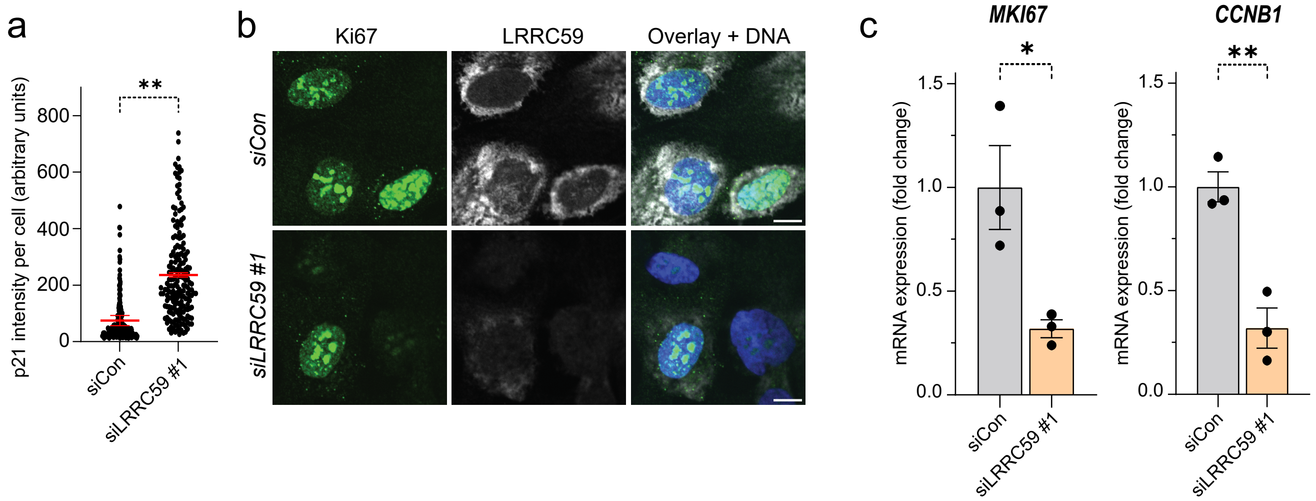

Supplementary figure 5. Consequences of LRRC59 depletion on cell proliferation
A LRRC59 depletion causes cell stress. Quantification of p21 intensity per cell after indicated treatments. Error bars: mean ± SEM of 3 independent experiments, each dot represents the mean intensity per cell; n = 231 (siCon); 192 cells (siLRRC59 #1). **P = 0.0012, two-tailed unpaired Student's t-test. B LRRC59 depletion reduces proliferation. Representative confocal images of control and LRRC59 depleted cells stained for ki67, LRRC59, and DNA (Hoechst). Scale bars, 10 µm; N = 3. C Decreased proliferation upon LRRC59 depletion. Quantification of *MKI67* and *CCNB1* mRNA levels normalized to *SF3A1*, in RPE1 cells treated with indicated siRNAs. Error bars: mean ± SEM of 3 independent experiments. *MKI67*: siCon vs. siLRRC59 #1, *P = 0.0301, two-tailed unpaired Student's t-test. *CCNB1*: siCon vs. siLRRC59 #1, **P = 0.0049, two-tailed unpaired Student's t-test. Source data are provided as a Source Data file.

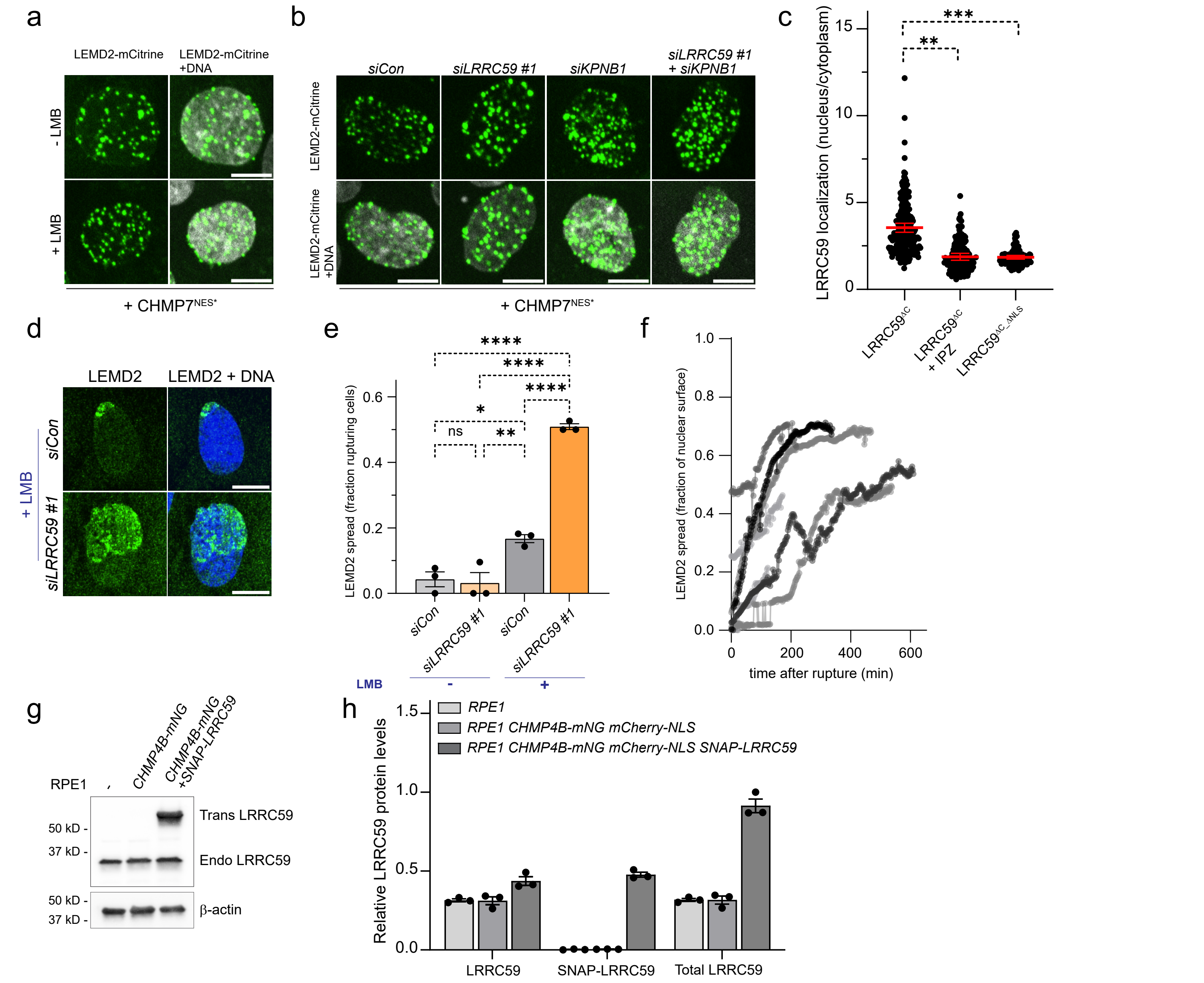

Supplementary figure 6. Mapping the effects of LRRC59 expression on LEMD2 NE spreading
A Stills from live-cell imaging of RPE1 LEMD2-APEX2-mCitrine cells treated with DOX to induce CHMP7^{NES*} expression and LMB. DNA was stained using SPY-650. Scale bars, 10 µm; N = 3. B Representative stills from live-cell imaging of RPE1 LEMD2-APEX2-mCitrine cells treated with indicated siRNAs and DOX to induce CHMP7^{NES*} expression. DNA was stained using SPY-650. Scale bars, 10 µm; N = 3. C Import of LRRC59 depends on its NLS. As in Figure 6G, graph shows quantification of LRRC59 localization. Error bars: mean ± SEM, 4 independent experiments, with dots representing individual cells; n = 237 (LRRC59$^{\Delta C}$), n = 155 (LRRC59$^{\Delta C}$+IPZ); 158 cells (LRRC59$^{\Delta C_\Delta NLS}$). LRRC59$^{\Delta C}$ vs LRRC59$^{\Delta C}$+IPZ, **P = 0.0011; LRRC59$^{\Delta C}$ vs LRRC59$^{\Delta C_\Delta NLS}$, ***P = 0.0006; LRRC59$^{\Delta C}$+IPZ vs LRRC59$^{\Delta C_\Delta NLS}$, NS, P = 0.9964. One-way ANOVA with Tukey's test. D Endogenous LEMD2 spread after perturbation of the LRRC59-KPNB1 and XPO1 axes. Representative confocal images of RPE1 cells treated with indicated siRNAs and LMB. Cells were stained for LEMD2 and DNA (Hoechst). Scale bars, 10 µm; N = 3. E LEMD2 spread triggered by combined disruption of LRRC59-KPNB1 and XPO1 axes. Live-cell imaging of RPE1 LEMD2-APEX2-mCitrine cells treated indicated siRNAs and LMB. Quantification of the fraction of nuclei exhibiting LEMD2 spread. Error bars: mean ± SEM, 3 independent experiments, with dots representing the mean per experiment; n = 32 (siCon, -LMB); 46 (siCon, +LMB); 61 (siLRRC59 #1, -LMB); 77 cells (siLRRC59 #1, +LMB). siCon -LMB vs LRRC59 #1 -LMB, NS, P = 0.979; siCon -LMB vs siCon +LMB, *P = 0.013; siCon -LMB vs siLRRC59 #1 +LMB; ****P < 0.0001; siCon +LMB vs siLRRC59 #1 - LMB, **P = 0.0078; siLRRC59 #1 -LMB vs siLRRC59 #1 +LMB, ****P < 0.0001; siCon +LMB vs siLRRC59 #1 +LMB, ****P < 0.0001. One-way ANOVA with Tukey's test. F As panel E, showing fraction of nuclear LEMD2 coverage over time in individual nuclei treated with siLRRC59 #1 and LMB. G Western blot showing endogenous LRRC59 and transgenic SNAP-LRRC59 protein levels in parent RPE1, RPE1 CHMP4B-LAP-mNG or RPE1 CHMP4B-LAP-mNG SNAP-LRRC59 cells. H Quantification of western blot shown in panel G showing relative levels of endogenous, transgenic SNAP-LRRC59, and total LRRC59. Source data are provided as a Source Data file.

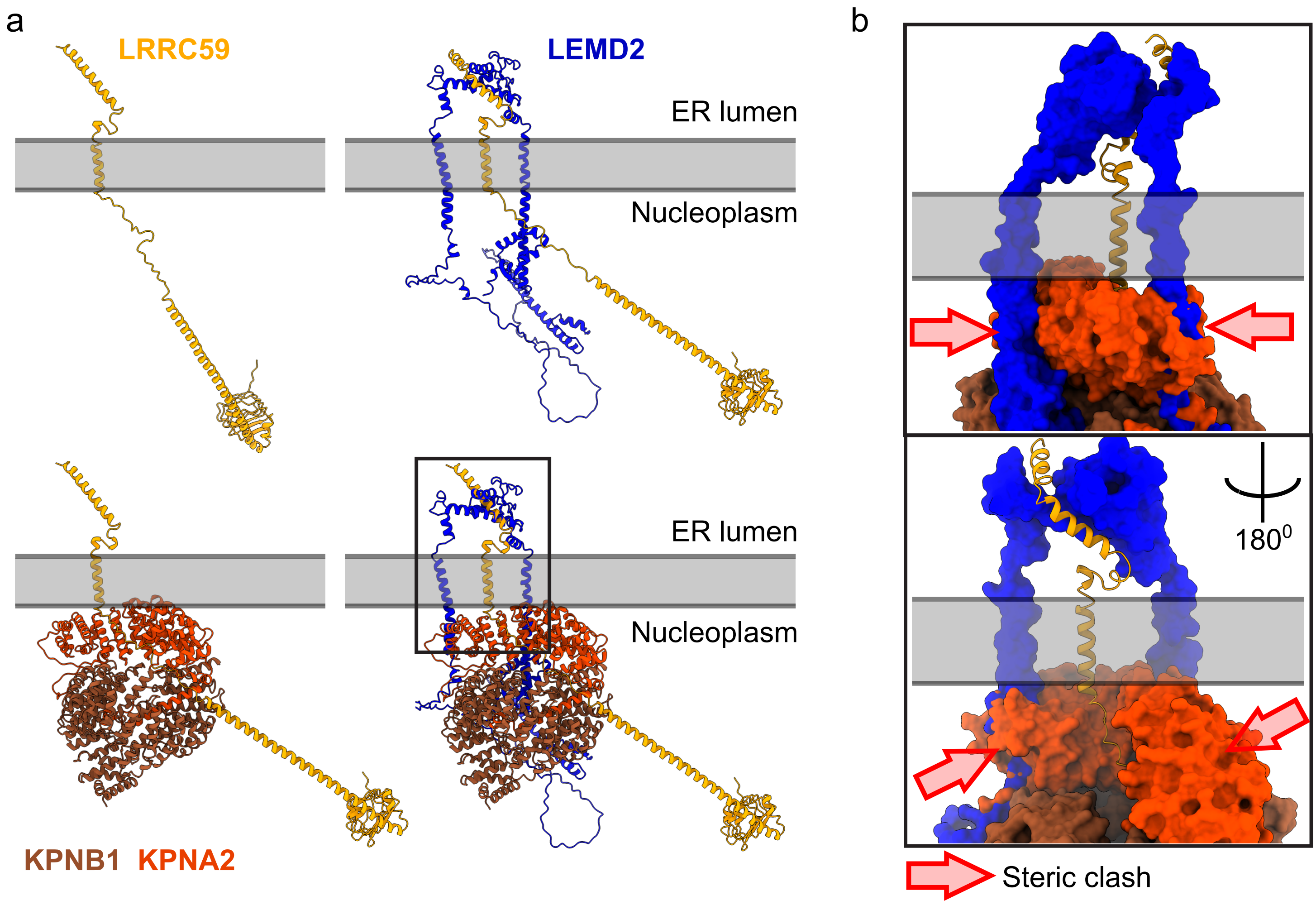

Supplementary figure 7. Alphafold modeling of LRRC59 interactions with LEMD2 and importins
A A ribbon representation of AlphaFold model of LRRC59 (yellow; top left), LRRC59 in association with LEMD2 (blue; top right), LRRC59 bound to KPNA2-KPNB1 (orange-brown; bottom left), or assembled into a quaternary LRRC59-LEMD2-KPNA2-KPNB1 complex. Lipid bilayer is in grey. B Close-up of boxed segment in panel A, bottom right. Space filling models for LEMD2 (blue) and KPNA2-KPNB1 (orange-brown) as well as LRRC59 (ribbon model) in two orientations showing spatial steric clashes between the long LEMD2 transmembrane helices (60-80 Å long) and the edges of the KPNA2-KPNB1 dimer (>50 Å diameter) indicated by red arrows.

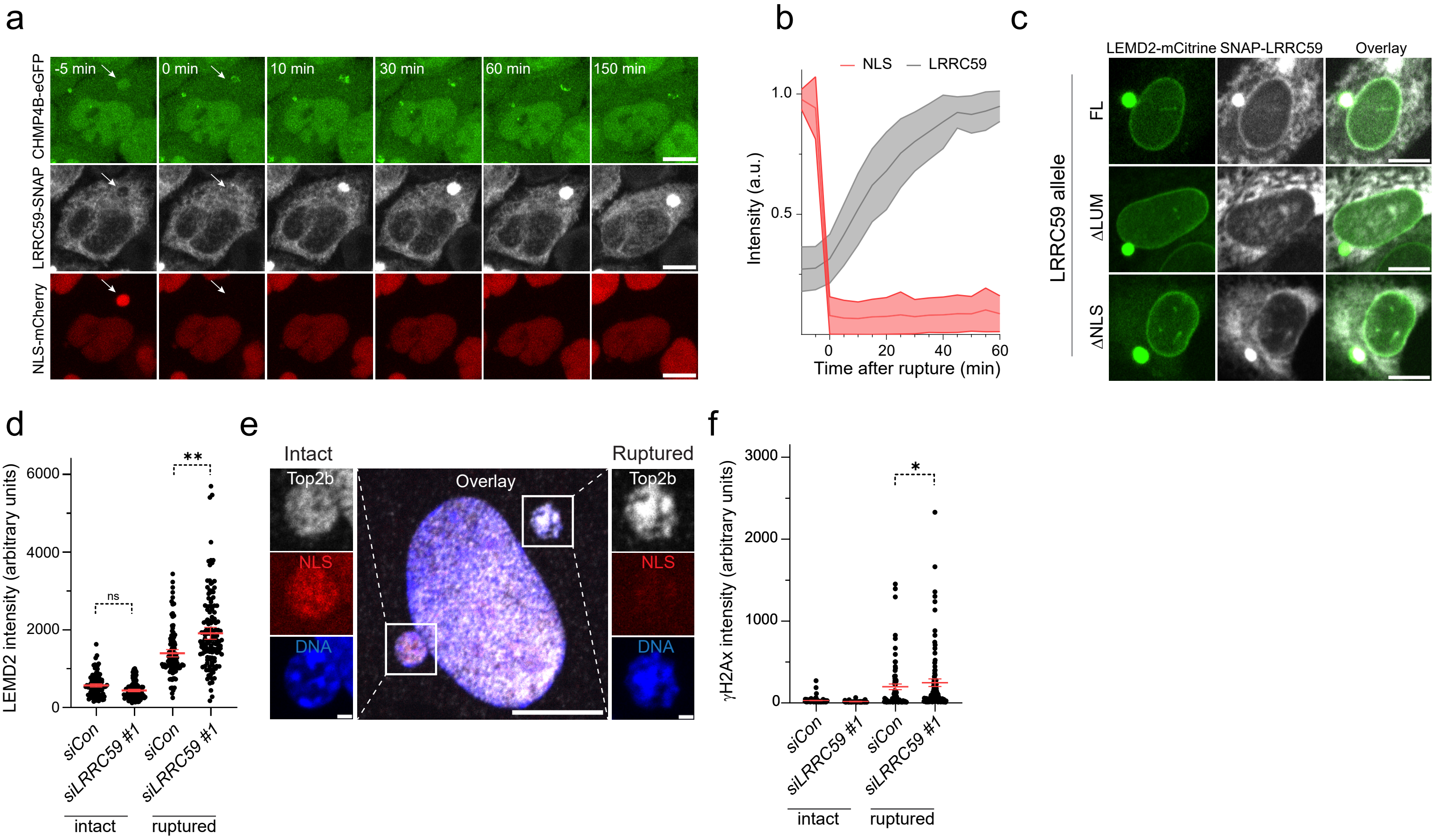

Supplementary figure 8. LRRC59 controls the repair and damage of ruptured micronuclei
A Stills from live-cell microscopy of HeLaK CHMP4B-LAP-mNG mCherry-NLS SNAP-LRRC59 cells at different timepoints during micronuclear rupture. LRRC59 was stained using SiR-SNAP. Arrows indicates MN. Scale bars, 10 μm; N = 3. B Quantification of SNAP-LRRC59 (gray) and mCherry-NLS (red) intensities at different time points during MN rupture, with t = 0 the first timepoint after rupture. Error bars: mean (lines) ± SD (bands), 3 independent experiments; n = 56 (LRRC59); 48 cells (NLS). C Representative confocal images of LRRC59 recruitment to ruptured MN in endogenous LRRC59-depleted RPE1 LEMD2-APEX2-mCitrine cells expressing siRNA #3-resistant SNAP-LRRC59 constructs. Scale bars, 10 μm; N = 3. D Quantification of LEMD2 mean intensity from RPE1 mCherry-NLS cells treated with indicated siRNAs and AZ3146. Error bars: mean ± SEM, 3 independent experiments, with dots representing the mean intensity per MN; n = 93 (siCon intact); 106 (siLRRC59 #1 intact); 98 (siCon ruptured); 133 cells (siLRRC59 #1 ruptured). siCon intact vs siLRRC59 #1 intact, NS, P = 0.3513; siCon ruptured vs siLRRC59 #1 ruptured, **P = 0.0046. One-way ANOVA with Fisher's LSD test. E Representative confocal images of RPE1 mCherry-NLS cells treated with AZ3146 and stained for Top2B. Inset shows intact or ruptured MN. Scale bars, 10 μm and 1 μm (inset); N = 4. F As panel D but now showing γH2Ax mean intensities. siCon ruptured vs siLRRC59 #1 ruptured, *P = 0.0211. Kruskal-Wallis with Dunn's test. Source data are provided as a Source Data file.

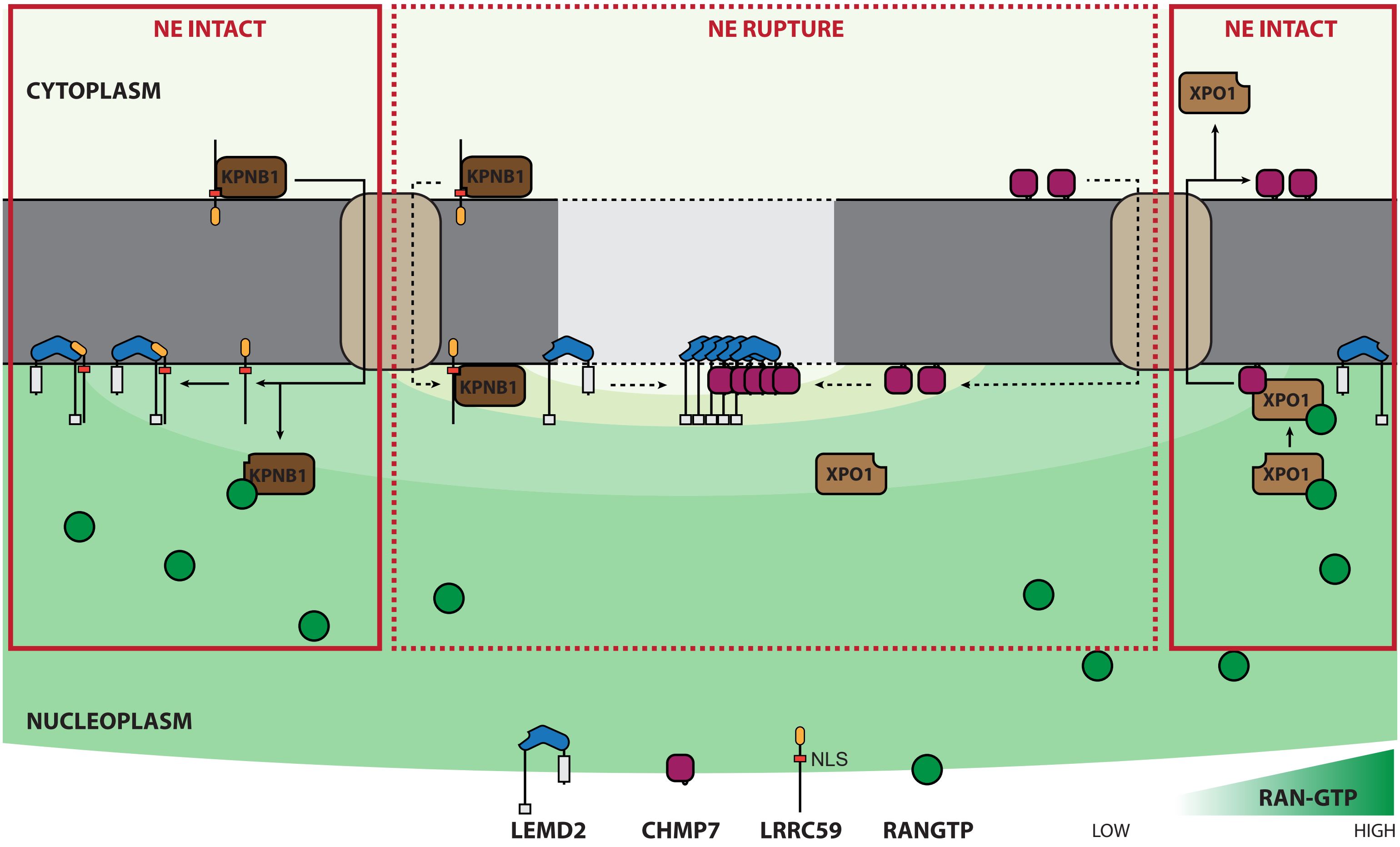

Supplementary figure 9. Model for LRRC59, KPNB1, XPO1 regulation of LEMD2-CHMP7 mediated NE repair
Model of the processes and interactions at NE rupture sites. Under intact NE conditions, CHMP7 remains in the ER through XPO1-RANGTP-facilitated nuclear export, ensuring its separation from the INM protein LEMD2 (right red box). LRRC59 is transported to the INM by KPNB1, where RANGTP dissociates it from KPNB1, allowing LRRC59 to interact with LEMD2 via its ER luminal domain (left red box). Upon NE rupture, the RAN gradient dissipates around the site of rupture, leading to CHMP7's dissociation from XPO1 and binding to LEMD2. Meanwhile, the disrupted RAN gradient prevents KPNB1 from dissociating from LRRC59 at the INM, causing steric interference that hindering LRRC59's binding to LEMD2. These events enable localized coalescence of LEMD2 and CHMP7 into a biomolecular condensate at the site of rupture, licensing polymerization of ESCRT-III filaments and thereby promoting NE repair (middle red box). After NE resealing, the RAN gradient's restoration triggers CHMP7's reassociation with XPO1-RANGTP, causing its release from LEMD2 and translocation back to the ER. Concurrently, LRRC59 separates from KPNB1-RANGTP, allowing it to bind LEMD2 again. Together this leads to the active breakdown of the LEMD2-CHMP7 condensate and reestablishment of the pre-rupture state.

Uncropped Western Blots

Supplementary Figure 1a

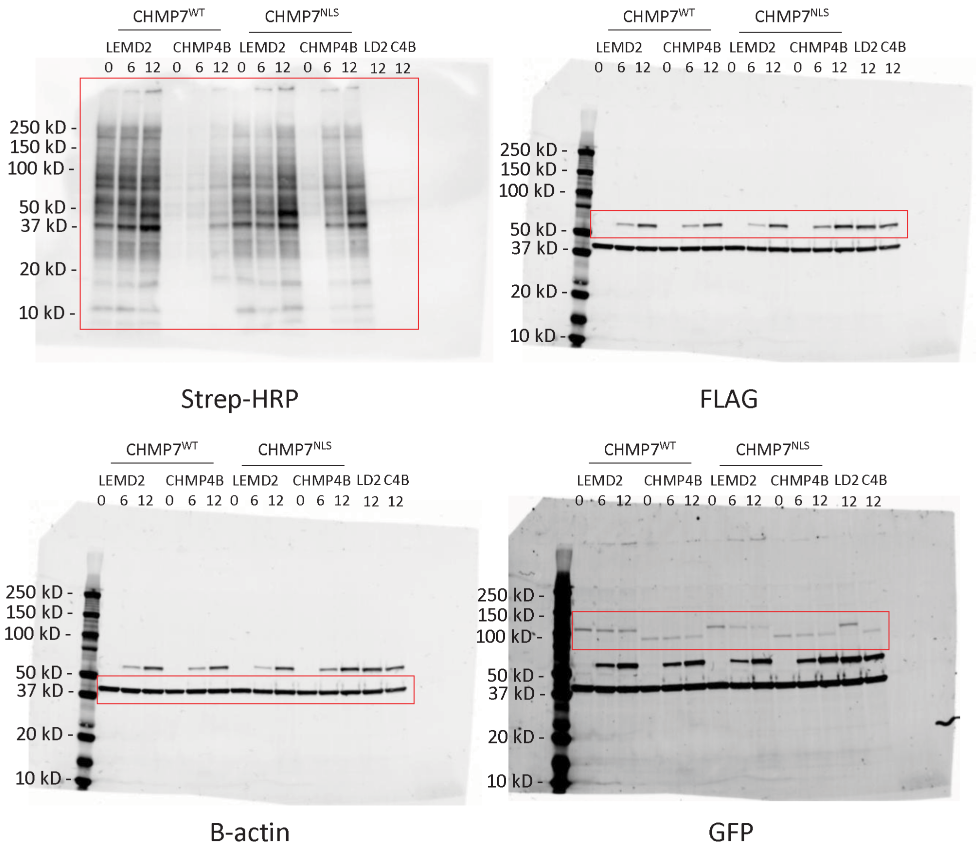

Supplementary Figure 1b

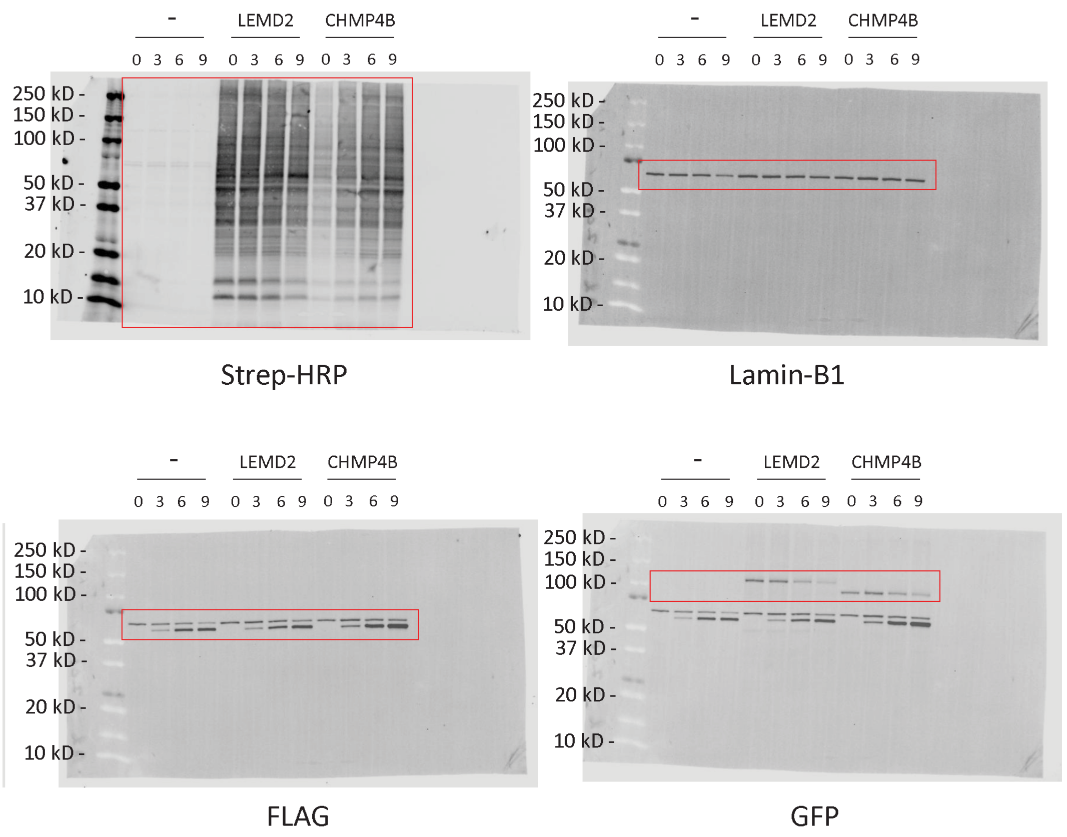

Supplementary Figure 10: Uncropped Western blots used in supplementary figures. Red rectangles indicate the cropped areas shown in the paper.

Uncropped Western Blots

Supplementary Figure 2a

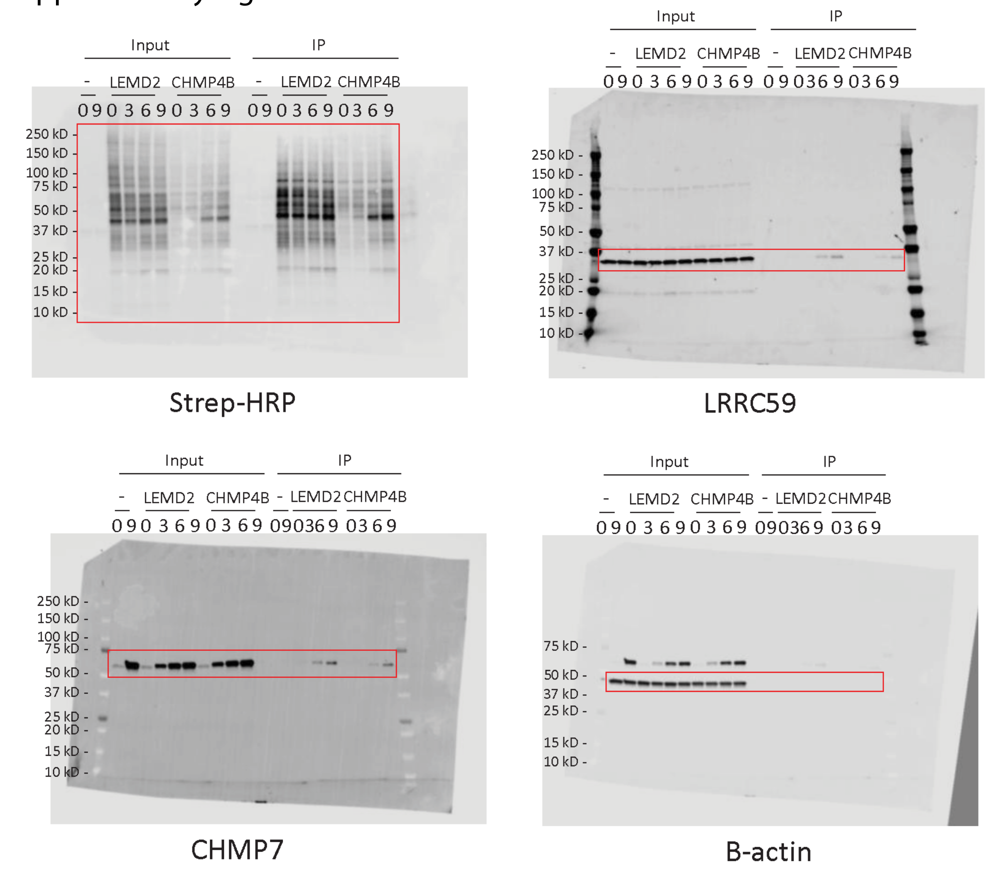

Supplementary Figure 2c

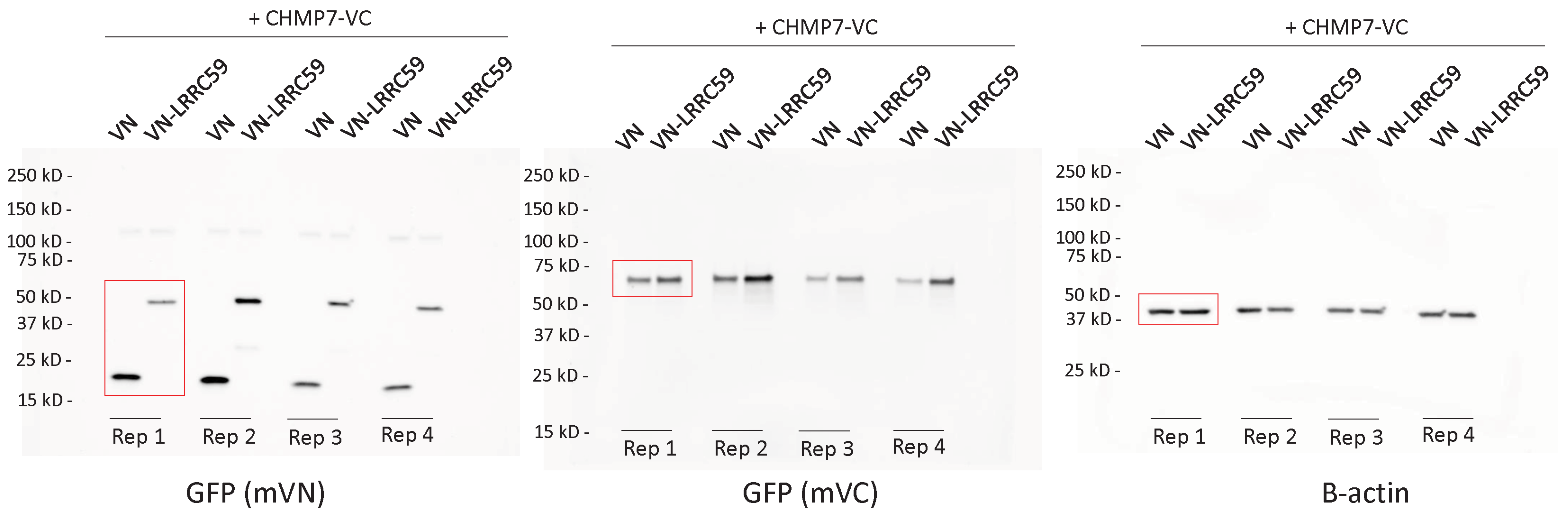

Supplementary Figure 2h

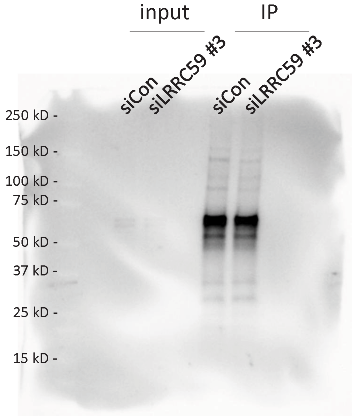

Supplementary Figure 10: Uncropped Western blots used in supplementary figures. Red rectangles indicate the cropped areas shown in the paper.

Uncropped Western Blots

Supplementary Figure 3a

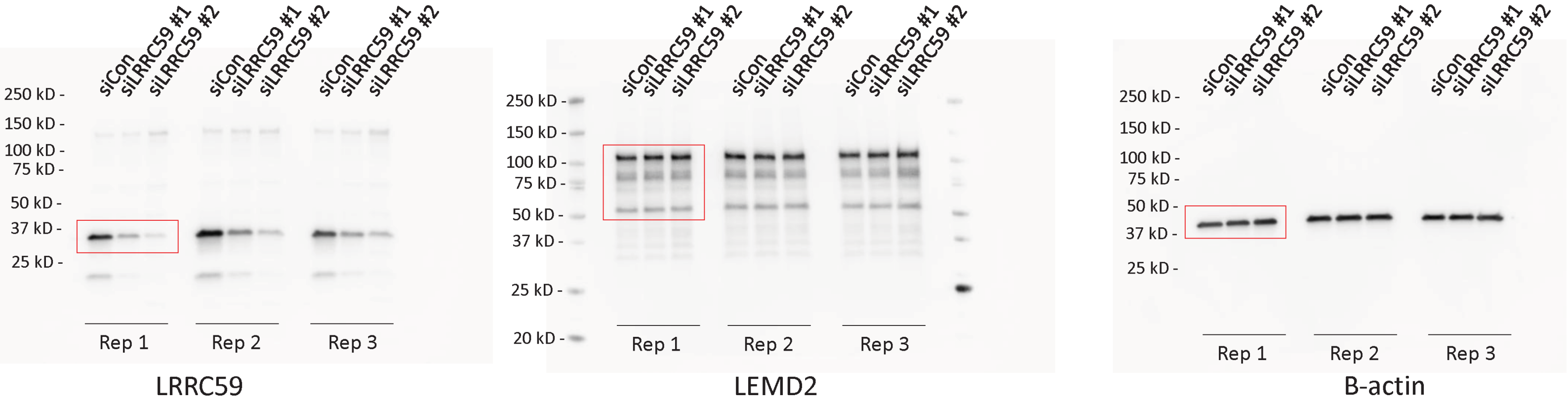

Supplementary Figure 6g

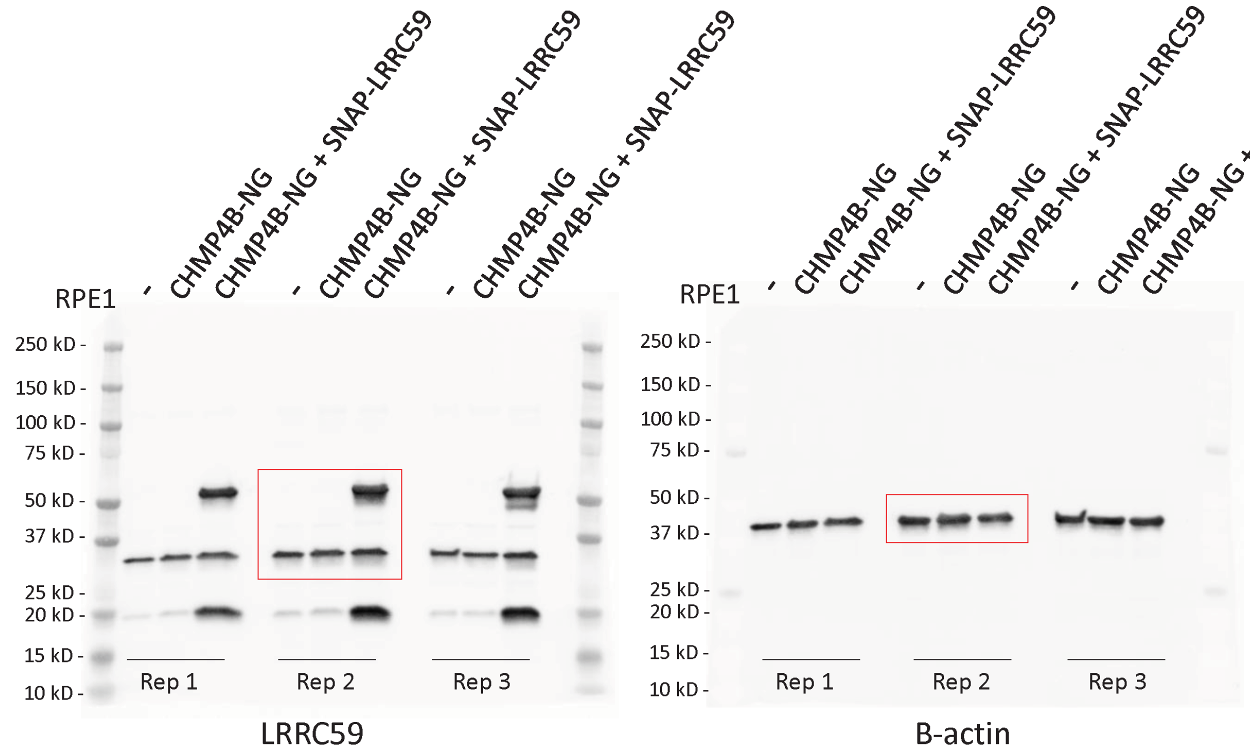

Supplementary Figure 10: Uncropped Western blots used in supplementary figures. Red rectangles indicate the cropped areas shown in the paper.

Supplementary Table 1. Stable cell lines used in this study.

#	Cell line	Transgene a	promoter	Transgene b	promoter	Transgene c	promoter
1	**RPE1**	*mCherry-NLS*	*EF1a*	*CHMP4B-LAPmNG-3HA*	*PGK*		
2	**RPE1**	*2xmRuby3-NES*	*EF1a*	*CHMP7-WT-FLAG*	*rTTA3*	*CHMP4B-LAP-APEX2-mCitrine*	*PGK*
3	**RPE1**	*2xmRuby3-NES*	*EF1a*	*CHMP7-NES*-FLAG*	*rTTA3*	*CHMP4B-LAP-APEX2-mCitrine*	*PGK*
4	**RPE1**	*2xmRuby3-NES*	*EF1a*	*CHMP7-NLS-FLAG*	*rTTA3*	*CHMP4B-LAP-APEX2-mCitrine*	*PGK*
5	**RPE1**	*2xmRuby3-NES*	*EF1a*	*CHMP7-WT-FLAG*	*rTTA3*	*LEMD2-APEX2-mCitrine*	*PGK*
6	**RPE1**	*2xmRuby3-NES*	*EF1a*	*CHMP7-NES*-FLAG*	*rTTA3*	*LEMD2-APEX2-mCitrine*	*PGK*
7	**RPE1**	*2xmRuby3-NES*	*EF1a*	*CHMP7-NLS-FLAG*	*rTTA3*	*LEMD2-APEX2-mCitrine*	*PGK*
8	**RPE1**	*HAeGFP-CHMP7*	*PGK*				
9	**RPE1**	*mCherry-NLS*	*EF1a*	*CHMP4B-LAPmNG-3HA*	*PGK*	*SNAP-LRRC59*	*PGK*
10	**RPE1**	*mCherry-NLS*	*EF1a*	*CHMP4B-LAPmNG-3HA*	*PGK*	*SNAP-LRRC59*	*rTTA3*
11	**HeLa K**	*mCherry-NLS*	*EF1a*	*CHMP4B-LAP-eGFP*	*Endo*	*SNAP-LRRC59*	*PGK*
12	**RPE1**	*2xmRuby3-NES*	*EF1a*	*CHMP7-NES*-FLAG*	*rTTA3*	*SNAP-LRRC59-FL (siRNAres)*	*PGK*
13	**RPE1**	*SNAP-LRRC59-FL (siRNAres)*	*PGK*	*LEMD2-APEX2-mCitrine*	*PGK*		
14	**RPE1**	*SNAP-LRRC59-DeltaLUM (siRNAres)*	*PGK*	*LEMD2-APEX2-mCitrine*	*PGK*		
15	**RPE1**	*SNAP-LRRC59-DeltaNLS (siRNAres)*	*PGK*	*LEMD2-APEX2-mCitrine*	*PGK*		
16	**RPE1**	*LRRC59-V5-TurboID*	*PGK*				
