# Supplementary material for: LRRC59 cooperates with nuclear transporters to restrain the nuclear envelope repair machinery and safeguard genome integrity
Source: Nat Commun. 2025 Dec 12;16:11211. doi: 10.1038/s41467-025-65994-4 (PMC12715200; doi:10.1038/s41467-025-65994-4)
Supplement: Supplementary file 2 — Description of Additional Supplementary Files [file 41467_2025_65994_MOESM2_ESM.pdf]

Description of Additional Supplementary Files

Supplementary Data 1 Cumulated data from all significantly enriched proteins identified in the label-free quantitative tandem mass spectrometry experiments performed in this study.

Supplementary Video 1
Live-cell imaging of RPE1 cells monitoring LEMD2-APEX2-mCitrine recruitment to the reforming NE following depletion of LRRC59 (anaphase onset at t = 0 min). SPY-650 is used as a DNA dye.

Supplementary Video 2
Live-cell imaging of RPE1 cells monitoring LEMD2-APEX2-mCitrine recruitment, residence time, and redistribution at the reforming NE during mitosis following depletion of LRRC59. SPY-650 is used as a DNA dye.

Supplementary Video 3
Live-cell imaging of RPE1 cells monitoring LEMD2-APEX2-mCitrine recruitment to NE ruptures (assessed by nuclear mRuby3–NES influx) following depletion of LRRC59 (rupture at t = 0 min).

Supplementary Video 4
Live-cell imaging of RPE1 cells monitoring eGFP-CHMP7 recruitment to NE ruptures following depletion of LRRC59 (rupture at t = 0 min).

Supplementary Video 5
Live-cell imaging of RPE1 cells monitoring CHMP4B-LAP-mNG recruitment to NE ruptures (assessed by nuclear mCherry-NLS efflux) following depletion of LRRC59 or CHMP2A (rupture at t = 0 min).

Supplementary Video 6
Live-cell imaging of RPE1 cells monitoring LEMD2-APEX2-mCitrine recruitment and spread at NE rupture sites following depletion of LRRC59 and treatment with the XPO1 inhibitor Leptomycin B (rupture at t = 0 min).

Supplementary Video 7
Live-cell imaging of RPE1 cells monitoring CHMP4B-LAP-eGFP recruitment and spread at NE rupture sites ruptures (assessed by nuclear mCherry-NLS efflux) following depletion of LRRC59 and treatment with the XPO1 inhibitor Leptomycin B (rupture at t = 0 min).

Supplementary Video 8
Live-cell imaging of RPE1 cells monitoring SNAP-LRRC59 recruitment to ruptured MN (assessed by mCherry-NLS micronuclear efflux) (rupture at t = 0 min).

Supplementary Video 9
Live-cell imaging of HelaK cells monitoring CHMP4B-LAP-mNG and SNAP-LRRC59 recruitment to ruptured MN (assessed by mCherry-NLS micronuclear efflux) (rupture at t = 0 min).

Supplementary Video 10

Live-cell imaging of RPE1 cells monitoring LEMD2-APEX2-mCitrine recruitment to ruptured MN following depletion of LRRC59 (rupture at t = 0 min).
