# Supplementary material for: LRRC59 cooperates with nuclear transporters to restrain the nuclear envelope repair machinery and safeguard genome integrity
Source: Nat Commun. 2025 Dec 12;16:11211. doi: 10.1038/s41467-025-65994-4 (PMC12715200; doi:10.1038/s41467-025-65994-4)
Supplement: Supplementary file 14 — Reporting Summary [file 41467_2025_65994_MOESM14_ESM.pdf]

nature portfolio

Corresponding author(s): Coen Campsteijn

Last updated by author(s): Sep 30, 2025

Reporting Summary

Nature Portfolio wishes to improve the reproducibility of the work that we publish. This form provides structure for consistency and transparency in reporting. For further information on Nature Portfolio policies, see our Editorial Policies and the Editorial Policy Checklist.

Statistics

For all statistical analyses, confirm that the following items are present in the figure legend, table legend, main text, or Methods section.

n/a	Confirmed	
☐	☒	The exact sample size (*n*) for each experimental group/condition, given as a discrete number and unit of measurement
☐	☒	A statement on whether measurements were taken from distinct samples or whether the same sample was measured repeatedly
☐	☒	The statistical test(s) used AND whether they are one- or two-sided<br>*Only common tests should be described solely by name; describe more complex techniques in the Methods section.*
☒	☐	A description of all covariates tested
☐	☒	A description of any assumptions or corrections, such as tests of normality and adjustment for multiple comparisons
☐	☒	A full description of the statistical parameters including central tendency (e.g. means) or other basic estimates (e.g. regression coefficient) AND variation (e.g. standard deviation) or associated estimates of uncertainty (e.g. confidence intervals)
☐	☒	For null hypothesis testing, the test statistic (e.g. *F*, *t*, *r*) with confidence intervals, effect sizes, degrees of freedom and *P* value noted<br>*Give P values as exact values whenever suitable.*
☒	☐	For Bayesian analysis, information on the choice of priors and Markov chain Monte Carlo settings
☒	☐	For hierarchical and complex designs, identification of the appropriate level for tests and full reporting of outcomes
☒	☐	Estimates of effect sizes (e.g. Cohen's *d*, Pearson's *r*), indicating how they were calculated

Our web collection on statistics for biologists contains articles on many of the points above.

Software and code

Policy information about availability of computer code

Data collection	softWoRx, Fusion, Chemidoc, Bio-Rad CFX Maestro
Data analysis	Fiji (1.54f), Cell Profiler (4.2.6), RStudio (4.1.2 and 4.4.1), Perseus (1.6.15), Cytoscape (3.10.2), Graphpad Prism (10), Microsoft Excel (365), AlphaFold3, Coot (1.1.15), Phenix (1.21.2), ChimeraX (1.8). Code available via Github (https://github.com/DeVosLab).

For manuscripts utilizing custom algorithms or software that are central to the research but not yet described in published literature, software must be made available to editors and reviewers. We strongly encourage code deposition in a community repository (e.g. GitHub). See the Nature Portfolio guidelines for submitting code & software for further information.

Data

Policy information about availability of data

All manuscripts must include a data availability statement. This statement should provide the following information, where applicable:

- Accession codes, unique identifiers, or web links for publicly available datasets
- A description of any restrictions on data availability
- For clinical datasets or third party data, please ensure that the statement adheres to our policy

All data shown and used to generate plots for this manuscript can be found in the source data file. Uncropped western blots are shown in Supplementary Fig 10. The proteomics data generated in this study have been deposited in public repository ProteomeXchange under PRIDE identifier PXD058192, and in included as Supplementary Data 1. All code is available via Github (https://github.com/DeVosLab). All other reagents and data will be made available upon request to the lead author

Reviewer access details
Log in to the PRIDE website using the following details:
Project accession: PXD058192
Token: Jw8W8K5mYAlt
Alternatively, reviewer can access the dataset by logging in to the PRIDE website using the following account details:
Username: reviewer_pxd058192@ebi.ac.uk
Password: f9uVGuXbGXCj

All datasets generated and/or analyzed during the current study are available from the corresponding author on request.

Research involving human participants, their data, or biological material

Policy information about studies with human participants or human data. See also policy information about sex, gender (identity/presentation), and sexual orientation and race, ethnicity and racism.

Reporting on sex and gender	*Use the terms sex (biological attribute) and gender (shaped by social and cultural circumstances) carefully in order to avoid confusing both terms. Indicate if findings apply to only one sex or gender; describe whether sex and gender were considered in study design; whether sex and/or gender was determined based on self-reporting or assigned and methods used. Provide in the source data disaggregated sex and gender data, where this information has been collected, and if consent has been obtained for sharing of individual-level data; provide overall numbers in this Reporting Summary. Please state if this information has not been collected. Report sex- and gender-based analyses where performed, justify reasons for lack of sex- and gender-based analysis.*
Reporting on race, ethnicity, or other socially relevant groupings	*Please specify the socially constructed or socially relevant categorization variable(s) used in your manuscript and explain why they were used. Please note that such variables should not be used as proxies for other socially constructed/relevant variables (for example, race or ethnicity should not be used as a proxy for socioeconomic status). Provide clear definitions of the relevant terms used, how they were provided (by the participants/respondents, the researchers, or third parties), and the method(s) used to classify people into the different categories (e.g. self-report, census or administrative data, social media data, etc.) Please provide details about how you controlled for confounding variables in your analyses.*
Population characteristics	*Describe the covariate-relevant population characteristics of the human research participants (e.g. age, genotypic information, past and current diagnosis and treatment categories). If you filled out the behavioural & social sciences study design questions and have nothing to add here, write "See above."*
Recruitment	*Describe how participants were recruited. Outline any potential self-selection bias or other biases that may be present and how these are likely to impact results.*
Ethics oversight	*Identify the organization(s) that approved the study protocol.*

Note that full information on the approval of the study protocol must also be provided in the manuscript.

Field-specific reporting

Please select the one below that is the best fit for your research. If you are not sure, read the appropriate sections before making your selection.

[x] Life sciences   [ ] Behavioural & social sciences   [ ] Ecological, evolutionary & environmental sciences

For a reference copy of the document with all sections, see nature.com/documents/nr-reporting-summary-flat.pdf

Life sciences study design

All studies must disclose on these points even when the disclosure is negative.

Sample size	In all experiments, trial runs were performed to determine what effect size was to be expected and how large and penetrant this effect was in the trial sample population. Based on this, we adapted the number and size of experiments to the expected effect size and the anticipated consistency between experiments. In support, the phenotypes described in this study are all highly penetrant and statistically significant.
Data exclusions	Outliers were identified using the robust regression and outlier removal (ROUT) test. Removal of these outliers did not affect the statistical significance of our findings, ensuring that the results presented are representative of the underlying trends in the data. No data was otherwise excluded for any reason
Replication	We reproduced all our findings and the exact number of replications of individual experiments are indicated in the text.
Randomization	For each experiment and treatment we used an appropriate experimental group that was non-treated, carrier treated, or treated with control siRNAs/chemicals as a reference to test for contribution of other covariates beyond the ones examined.
Blinding	Data acquisition for all live-cell microscopy was effectively blinded because phenotypes were not visible at the starting time of acquisition. Additionally, for fixed imaging, cells were selected for imaging based on channels (e.g. DAPI) other than the channel used for the experimental variable. For high-throughput fixed microscopy, imaging field selection was random. Furthermore, data analysis was frequently done by other

multiple people, with only non-descriptive names (e.g. field number) as guide. This approach prevented subconscious biases during analysis of imaging data. Blinding for western blots was not relevant as the phenotype was only revealed after completion of the experimental pipeline.

Reporting for specific materials, systems and methods

We require information from authors about some types of materials, experimental systems and methods used in many studies. Here, indicate whether each material, system or method listed is relevant to your study. If you are not sure if a list item applies to your research, read the appropriate section before selecting a response.

Materials & experimental systems

n/a	Involved in the study	
[ ]	[x]	Antibodies
[ ]	[x]	Eukaryotic cell lines
[x]	[ ]	Palaeontology and archaeology
[x]	[ ]	Animals and other organisms
[x]	[ ]	Clinical data
[x]	[ ]	Dual use research of concern
[x]	[ ]	Plants

Methods

n/a	Involved in the study	
[x]	[ ]	ChIP-seq
[x]	[ ]	Flow cytometry
[x]	[ ]	MRI-based neuroimaging

Antibodies

Antibodies used

1. mouse anti-CHMP7 (Abnova H00091782-B01P; IF, 1:100; PLA, 1:200; lot m5041)
2. rabbit anti-CHMP7 (Proteintech 16424-1-AP; WB, 1:1000; lot 00039615)
3. mouse anti-FLAG (Sigma-Aldrich, F1804; PLA, 1:750; WB, 1:2000; lot SLBS3530V)
4. rabbit anti-LRRC59 (Sigma-Aldrich, HPA030829; IF, 1:300; PLA, 1:250; WB, 1:1000; lot A105279)
5. mouse anti-b-actin (Proteintech, 66009-1-Ig; WB, 1:10000; lot 10004156 & 10025459)
6. rabbit anti-LaminB1 (Abcam, ab16048; WB, 1:1000; lot GR3459550-1)
7. rabbit anti-LEMD2 (Sigma-Aldrich, HPA017340; WB, 1:1000; IF, 1:200; lot 000007624)
8. mouse anti-Top2b (SCBT, sc-25330; IF, 1:100; lot I1817)
9. rabbit anti-GFP (Abcam, ab32146; WB, 1:5000; lot GR253725-25)
10. mouse anti-eGFP (Roche, 11814460001; IF, 1:750; PLA, 1:300; WB, 1:1000; lot 19958500)
11. mouse anti-gH2Ax (Millipore, 05-636; IF, 1:500; lot 3761799)
12. Streptavidin-AF647 (ThermoFisher, s21374; IF, 1:500; lot 1924460)
13. Streptavidin-HRP (ThermoFisher, s911; WB, 1:5000; lot 1880067)
14. rabbit anti-SNAPtag (ThermoFisher, CAB4255; WB, 1:1000; lot XC343633)
15. mouse anti-LBR (Abcam, ab232731; WB, 1:1000; lot GR3353170-6)
16. mouse anti-Lamin A/C (SCBT, sc-7292; WB, 1:500; lot H0720)
17. mouse anti-p21/CDKN1A (SCBT, sc-6246; IF, 1:200; lot B2820)
18. mouse anti-Ki67 (SCBT, sc-23900; IF, 1:200; lot F0118)

Validation

Primary antibody validation was performed using siRNA-mediated depletion of target proteins in western blotting (1, 2, 4, 6, 7, 16) and immunofluorescence staining (1, 4, 6, 7, 15, 16). In addition, several antibodies were validated by expression of transgenic fusion proteins, assessed by western blotting or immunofluorescence (3, 4, 7, 9, 10, 14). Secondary validation criteria included detection of the expected molecular weight in western blot assays (2, 3, 4, 5, 6, 7, 9, 10, 14, 15, 16) and localization patterns consistent with those reported for other published antibodies. Furthermore, many antibodies (3, 5, 6, 8, 9, 10, 11, 12, 13, 14, 15, 16, 17, 18) have been independently validated by commercial suppliers or other laboratories for the applications described here.

Secondary antibodies included anti-mouse- and anti-rabbit -Alexa488, -Alexa568, and -Alexa647 (all cross-adsorbed Molecular Probes), -IRDye680, -IRDye800 (LI-COR), and -HRP (Jackson) conjugates that have all been extensively characterized by the companies and numerous labs.

Eukaryotic cell lines

Policy information about cell lines and Sex and Gender in Research

Cell line source(s)	Cell lines were acquired from ATCC (RPE1; CRL-4000) or are commonly used cell lines (HeLa "Kyoto", HEK293T) obtained from scientific collaborators (D. Gerlich and A. Hyman).
Authentication	Genotyping has been performed for the cell lines used in this study
Mycoplasma contamination	All cell lines are routinely tested for mycoplasma contamination and all tested negative for mycoplasma
Commonly misidentified lines (See ICLAC register)	No commonly misidentified lines were used in this study

Plants

Seed stocks	*Report on the source of all seed stocks or other plant material used. If applicable, state the seed stock centre and catalogue number. If plant specimens were collected from the field, describe the collection location, date and sampling procedures.*
Novel plant genotypes	*Describe the methods by which all novel plant genotypes were produced. This includes those generated by transgenic approaches, gene editing, chemical/radiation-based mutagenesis and hybridization. For transgenic lines, describe the transformation method, the number of independent lines analyzed and the generation upon which experiments were performed. For gene-edited lines, describe the editor used, the endogenous sequence targeted for editing, the targeting guide RNA sequence (if applicable) and how the editor was applied.*
Authentication	*Describe any authentication procedures for each seed stock used or novel genotype generated. Describe any experiments used to assess the effect of a mutation and, where applicable, how potential secondary effects (e.g. second site T-DNA insertions, mosiacism, off-target gene editing) were examined.*
